# Alfred Russel Wallace’s legacy: an interdisciplinary conception of evolution in space and time

**DOI:** 10.1038/s44185-023-00010-w

**Published:** 2023-01-06

**Authors:** Joaquín Hortal, José Alexandre F. Diniz-Filho, Martyn E. Y. Low, Alycia L. Stigall, Darren C. J. Yeo

**Affiliations:** 1 Department of Biogeography and Global Change, Museo Nacional de Ciencias Naturales (MNCN-CSIC), Madrid, Spain; 2 Departamento de Ecologia, Instituto de Ciências Biológicas, Universidade Federal de Goiás (UFG), Goiânia, GO Brazil; 3 Lee Kong Chian Natural History Museum, Faculty of Science, National University of Singapore, Singapore, Republic of Singapore; 4 Department of Earth and Planetary Science, University of Tennessee, Knoxville, TN USA; 5 Department of Biological Sciences, National University of Singapore, Singapore, Republic of Singapore

“Wallace (1852 and in general) was right.”

Meiri 2018^1^, p.4.

Imagine an undaunted explorer with the scientific intellect of Leonardo Da Vinci, Marie Curie, or Albert Einstein. Someone for whom a life of great adventure (and catastrophic misfortune) went hand in hand with seminal scientific discoveries. Alfred Russel Wallace (1823–1913) was just such a person in biodiversity science. He was an explorer, prolific collector, co-discoverer of key laws of ecology and evolution such as the principle of natural selection (and perhaps others as we will see below), and the true founder of the vibrant multidisciplinary science of biogeography. He deserves both a central position in the scientific hall of fame, as well as a multi-season series mixing action and intrigue on a streaming platform depicting his long and adventurous life.

In the short space of an editorial, it is impossible to account for Wallace’s multiple major contributions, the particularities of his character and the oddities that his curiosity and critical spirit led him to defend. A comprehensive account of his prolific works and influence can be accessed at https://wallacefund.myspecies.info/content/scientific-legacy. Here, we provide a short semblance of his life and legacy through three key episodes of his long life, honoring his adventures, discoveries, and theoretical advances as he amassed specimens and data in the Amazon (which were all virtually lost), where having learned his lessons tried again in the Malay Archipelago (not without hardships of its own), and fought his lasts crusades in the scientific arena.

## Explorer of the Amazon

In April 1848, a 25-year-old Alfred Russel Wallace embarked to the Amazonian Pará with Henry Walter Bates, another enthusiastic naturalist and entomologist, with the idea of earning their living by collecting as many specimens of animals and plants as possible for the growing collections of British museums and private collectors that, at the time, were enthusiastic for the natural history of the tropical regions. Wallace was a self-taught naturalist with no formal education, coming from a family of limited resources but with a keen interest in the natural sciences grown during his walks around the British Midlands and nurtured with his readings of the most relevant scientific literature of the time. Among other books, he was particularly inspired by C. Lyell’s *Principles of Geology*, R. Chambers’s *Vestiges of the Natural History of Creation*, C. Darwin’s *The Voyage of the Beagle* and W.H. Edwards’s *A Voyage up the Amazon*, the volume that ultimately inspired him and Bates to organize a collecting expedition to the luxuriant forests of tropical South America.

Wallace spent four years (1848–1852) traveling throughout the Amazon basin (Bates remained eleven), collecting specimens and making numerous observations of the biodiversity, the geography, and its peoples. He was mainly based at Pará, making frequent expeditions to other regions of the Amazon, including a 6-month solo expedition to the Río Negro after splitting with Bates. The observations he made during these years are beautifully narrated in Wallace’s *Travels on the Amazon and Rio Negro*^2^ and established the bases of some of his most important scientific contributions to (bio)geography, ecology and evolution. These include the first biogeographic regionalization ever proposed, based on the hypothesis that major rivers acted as barriers, and whose borders still hold up in modern analyses of many taxa^3^, but not necessarily for others^4^; and his classification of rivers according to the content of their waters, which determine the distribution and diversity of fish^5^. Like Humboldt^6^ and Darwin^7^, Wallace also made detailed observations of bird plumage variations at the individual level that sustained his ideas about ecological adaptation, sexual selection, and biogeographical trends^8^ and which are currently reinvigorated by current macroecology and macroevolution^9,10^.

## From misfortune to the Malay Archipelago

Wallace and his collections went aboard the ship *Helen* on July 12, 1852 for the voyage home. On August 6, Wallace was informed by the captain that the ship was on fire. All souls were safely transferred into small boats, but Wallace lost everything except for some papers. He and his shipmates were rescued after bobbing about for ten days^11^. It is thus not surprising that Wallace exclaimed, “Oh, glorious day!” on the first day of October 1852 upon reaching England from South America^11^. Three days later, Wallace was present at an Entomological Society meeting^12^. The day after the meeting he wrote an associate saying that “fifty times since I left Pará have I vowed, if I once reached England, never to trust myself more on the ocean. But good resolutions soon fade …”^11^.

Resolve faded less than eighteen months later, and Wallace was Asia-bound—specifically to the island of Singapore at the heart of Southeast Asia. His close study of the Insect and Bird Departments of the British Museum led him to decide that Singapore should be a new starting point for his natural history collections^13^. Singapore enthused and amused Wallace, and he made full use of the island’s Goldilocks qualities—remote enough that specimens and information were in demand, but connected enough to England to ensure timely conveyance of letters and specimens; and with forests pristine enough that the richness of beetles still surprised Wallace (an expert of the group), but sufficiently disturbed to facilitate his collecting^11,14^. The island would fulfill more than the practical aspects of natural history specimen collecting; Wallace compared his Singapore specimens constantly with material from elsewhere in his taxonomic papers^15^. The island was also a yardstick he would use to compare other localities against in broader terms of species diversity and eventually biogeography, aesthetic beauty, or even the availability of fruits^11^.

For eight years (1855–1862), Wallace explored the Malay Archipelago, including modern-day Singapore, Malaysia, and Indonesia, traveling over 22,000 km and collecting 125,660 insect specimens^16^. The observations made during these field expeditions led him to propose the famous “Wallace line”, a geographical barrier separating Asian and Australian biota that we now know comes from the distinct evolutionary processes caused by long-term geological and sea-level dynamics on the isolation of the biotas of the continental and oceanic islands of the Malay Archipelago^17–20^.

It was during this period that Wallace wrote his ideas on evolution for the first time, in two key essays. First, during a long stay at Sarawak, in Borneo, he set up the basis of modern biogeographical ideas on the distribution of species, genera, and higher taxa and—importantly—on the origin of new species in the seminal “Sarawak paper”^21^. Later at Ternate, in the Maluku Archipelago, he described the mechanism of natural selection in the famous “Ternate paper”^22^. He sent this essay to Darwin, asking him to present it to the British scientific community, laying the foundations of, perhaps, the greatest scientific revolution of the 19th Century: the theory of evolution via natural selection.

## The setup of the ecological and spatial components of evolution

Wallace is recognized as the co-discoverer of the natural selection principle, although for a long time he was relegated to a supporting role, often regarded as the important but secondary “trigger” that prompted Darwin to reveal his theory to the world. Darwin worked on his theory of “descent with modifications” by natural selection for more than 20 years until he received Wallace’s Ternate paper in early 1858. This put in motion a series of events that led to the joint presentation of manuscripts by Darwin and Wallace in the Linnean Society of London on July 1, 1858 and, about a year later, to the publication of *The Origin of Species* by Darwin^23^. The letters written prior to this correspondence and Wallace’s “Sarawak paper”, the events that unfolded after the arrival of Wallace’s essay to Darwin’s hands and the role played by Charles Lyell, Joseph Dalton Hooker and other British academics have been the subject of very detailed analyses of who wrote what and when^24,25^. But the truth is that Wallace lacked the social status of Darwin and needed his assistance to fully promote his ideas. Wallace never complained about priority and accepted his role in the early establishment of the first steps of evolutionary theory. When Wallace summarized his own view of evolutionary theory much later, in 1889, he entitled it *Darwinism*^26^, reflecting his recognition of Darwin’s priority in the discovery of natural selection.

Wallace continued working to become a recognized naturalist and initiated several new research fields, in particular in evolutionary biogeography. Unfortunately, he was discredited among his contemporaries due to his disagreements with Darwin about sexual selection playing a distinct evolutionary role independent from natural selection, as well as by the unorthodox character of some of his later works^27^. Such discredit was in many ways unfair, as Darwin’s hypothesis of the independent role of sexual selection was not widely accepted until the early 20th century^28^, while Wallace’s proposed mechanisms for the origin of sexual dimorphism based on the natural selection are currently recognized as valid^29^. Nonetheless, later in his life, Wallace embraced more pluralistic views of evolution and was seduced by teleological and metaphysical ideas—although in his mind he thought he was in all cases widening the role of natural selection^27^. He also became involved in social activism—becoming a socialist, and wrote about different aspects of culture and society, always under the perspective of evolution, progress, and development. To be fair, Wallace was a man of his time, immersed in his contemporary cultural and intellectual environments and being right and wrong about different issues (which include moderate views on racism, colonialism, and eurocentrism, and an embarrassing involvement in an early version of the anti-vaccine movement).

The truly everlasting legacy of Wallace comes from his original and creative thoughts about evolution, and his innovative approach to biogeography—which was actually the foundation of his evolutionary theory^27^. It is in this latter field where Wallace is more widely recognized today. Wallace proposed the existence of biogeographic regions with distinct faunas arising from historical evolutionary and geological processes. Not only did he get the processes right (although our knowledge on them has changed a lot since his time), but the borders and regions he proposed frequently match recent analyses based on large phylogenies^30,31^ or modern hypotheses about the origin of biogeographical barriers^32,33^. Today, biogeographic regionalization is a well-established discipline that is helping us understand the spatial organization and evolutionary origin of the diversification of many terrestrial and marine groups^4,34,35^.

His knowledge of broad-scale geographical patterns of diversity provided Wallace with the basis for his most innovative -and now widely proved- views of evolutionary dynamics. A careful reading of the Sarawak^21^ and Ternate papers^22^ reveals that Wallace recognized many biogeographical patterns and interpreted them from an evolutionary perspective. Wallace was an early proponent of the importance of allopatric speciation by geographic isolation, vicariance, and geologic history in shaping both individual species formation and geographic distributions^14,21^. Although he later moderated his views to match Darwin’s dispersalist paradigm^36^, his earlier work focused strongly (and correctly) on the significance of processes fostering population isolation and the derivation of new species coupled in both space and time with ancestral lineages including fossil evidence^37^. Significantly, he also theorized about the origin of these spatial patterns in the context of the origin of species—which were not that clear in Darwin’s thoughts^28^. Darwin conceptualized natural selection as an adaptive force increasing the fit between phenotypes and environment, leading to the anagenetic transformation of species into new ones. Whereas Wallace already acknowledged that the geographic context of the processes leads to the origination of species: his early hypotheses about the role of large rivers in the origination of the Amazonian biota^2^ implies that divergent populations evolving in different areas split into new species, leading to cladogenesis and increasing overall biodiversity. In this context, Wallace also anticipated several aspects of the “biological species concept” proposed by Ernst Mayr in the 1940s.

Wallace synthesized his biogeographical conception of evolutionary processes in the two volumes of *The Geographic Distributions of Animals*^37^ and later on *Island Life*^38^ (see ref. ^39^). The visionary character of these ideas can be exemplified by the way he anticipated Tobler’s 1970 first law of geography: “everything is related to everything else, but near things are more related than distant things”^40^. Well over a century ahead of this time, and despite (or perhaps because of) suffering from fever in the jungle in Sarawak, Wallace merged the principle of spatial dependence enunciated by Tobler’s law with evolutionary processes in a much more elegant way, writing that “every species has come into existence coincident both in time and space with a pre-existing closely allied species”^21^.

## Concluding remarks

Alfred Russel Wallace was a brilliant scientist, and a prolific writer, who wrote 508 scientific papers and 22 books, totaling over 10,000 pages^16^. But despite his many scientific contributions and far-reaching influence, 200 years after his birth Wallace’s name is seldom known beyond those that work directly on ecology and evolution. Wallace’s views on the spatial and ecological patterns of diversification were complementary to—and advanced beyond—a large amount of evidence on evolution accumulated by Darwin over decades, and the theoretical body they constructed remains as the basis of modern evolutionary theory. Thus, that Wallace has remained under the shadow of his peer Charles Darwin instead of being well-known among the general public is an injustice that Darwin himself would regret, and the people and institutions dedicated to disseminating knowledge about biodiversity and familiar with his story have been fighting to correct. More importantly, the correction should be one of shining a light not on Wallace as a Victorian explorer and scientist, but on his interdisciplinarity, his never losing sight of the rainforest for the trees, his sense of wonderment for nature, and crucially in reminding us that sometimes we need to get out of our laboratories and our silos and see the World for ourselves.
